# Hospital Notes

**Published:** 1895-06

**Authors:** 


### Hospital Note.

A COMPETITIVE examination of candidates for positions as house physicians in the Erie County Hospital will be held at the hospital, Buffalo, N. Y., on May 28, 1895, at 3.30 o'clock P. M. There are three vacancies to be filled June 1st. All applications should be addressed to Dr. E. J. Gilray, medical superintendent of the hospital.

